# Five-Year Trends in Multidrug- and Extensively Drug-Resistant Enterobacterales Causing Pediatric Urinary Infections in Southern Türkiye: A Surveillance Report

**DOI:** 10.5152/eurasianjmed.2026.251244

**Published:** 2026-03-17

**Authors:** Şinasi Karvar, Sibel Gümüş, Elçin Kal Çakmaklıoğulları

**Affiliations:** 1 Department of Medical Microbiology, Alanya Alaaddin Keykubat University Faculty of Medicine, Antalya, Türkiye

**Keywords:** Antibiotic resistance, drug resistance, pediatrics, urinary tract infection

## Abstract

**Background:**

This study aimed to assess species distribution, antimicrobial susceptibility, and temporal trends in multidrug-resistant (MDR), extensively drug-resistant (XDR), and extended-spectrum beta-lactamase (ESBL)–suspect phenotypes among pediatric urinary *Enterobacterales* isolates at a tertiary center.

**Methods:**

The authors retrospectively reviewed positive urine cultures from patients younger than 18 years between January 2020 and August 2025. Species identification and antimicrobial susceptibility testing (AST) were performed primarily with VITEK 2, supplemented with biochemical tests and disk diffusion when needed. Multidrug-resistant (MDR), XDR, and pandrug-resistant (PDR) phenotypes were classified according to standard definitions. The ESBL-suspect phenotype was defined as concurrent non-susceptibility to at least one third-generation cephalosporin and to aztreonam. Trend analyses were limited to 2020-2024.

**Results:**

Among 2889 *Enterobacterales* isolates, species distribution was as follows: *Escherichia coli* 69.4%, *Klebsiella* spp. 20.6%, *Proteus* spp. 5.6%, *Enterobacter* spp. 2.1%, others 1.9%. Over time, MDR increased in *E. coli* (37.7% to 57.8%), with parallel increases in ESBL-suspect and XDR phenotypes. In *Klebsiella* spp., XDR rose from 6.3% to 11.4%. Pandrug-resistant phenotype was detected only in 3 *Klebsiella* isolates and not in other species. Susceptibility to carbapenems and amikacin was largely preserved throughout the study. In *E. coli*, nitrofurantoin and fosfomycin retained high activity, whereas oral options were limited for *Klebsiella* spp.

**Conclusion:**

The rising MDR burden in pediatric *Enterobacterales* and the increase in XDR among *Klebsiella* spp. warrant regular revision of empiric therapy protocols. Preserved susceptibility to carbapenems and amikacin remains important for severe cases. Age-aware, time-trend surveillance that couples species distribution with resistance trajectories can strengthen antimicrobial stewardship.

**Main Points**

- A 5-year surveillance of 2889 pediatric *Enterobacterales* urinary isolates revealed a significant increase in multidrug- and extensively drug-resistant (MDR/XDR) phenotypes, particularly among *Escherichia coli* and *Klebsiella* species.
- Despite this rise, amikacin, carbapenems, nitrofurantoin, and fosfomycin retained high activity and remain the most reliable therapeutic options for pediatric urinary infections.
- The growing MDR/XDR burden underscores the urgent need for regular local resistance monitoring and integration of pediatric data into national antimicrobial stewardship programs.
- European Committee on Antimicrobial Susceptibility Testing–based statistically robust trend analysis (Cochran–Armitage and weighted logistic regression) ensures comparability with international AMR surveillance data.

## Introduction

Urinary tract infections are clinically important in children because of their high frequency at presentation and the need for treatment.^1^^,^^2^ Pathogens within the Enterobacterales group account for a substantial proportion of these infections and directly influence empiric therapy choices.^2^^,^^3^ Antimicrobial resistance patterns are among the principal determinants of clinical management outcomes; in particular, extended-spectrum beta-lactamase (ESBL) production and the multidrug-resistant (MDR),extensively drug-resistant (XDR), and pandrug-resistant (PDR) phenotypes can restrict available treatments.^2^^,^^4^^,^^5^

In the pediatric population, studies that concurrently evaluate species distribution, antibiotic susceptibility profiles, and temporal changes in multiple resistance phenotypes within the same cohort remain limited.^2^^,^^4^^,^^5^ Much of the existing literature either focuses on a single phenotype or assesses a limited set of antibiotic classes.^2^^,^^5^ In routine clinical practice, however, updating empiric treatment pathways requires simultaneous surveillance of species distribution, resistance phenotypes, and susceptibility to selected key agents.^6^^,^^7^

Accordingly, this study aims to evaluate species distribution, antibiotic susceptibility profiles, and the ESBL-suspect, MDR, XDR, and PDR phenotypes, together with their temporal trends, in a large, single-center pediatric cohort covering 2020-2025. This framework is intended to provide an updated basis for evidence-informed and regularly scheduled revisions of empiric therapy decisions.

## Material and Methods

### Study Design and Population

This retrospective cross-sectional study covers positive urine culture results from pediatric patients aged 0-18 years who presented to Training and Research Hospital between January 2020 and August 2025. During the study period, 3359 positive urine cultures were evaluated. Applying a single isolate per patient rule, repeat isolations were excluded, and the analytic cohort, comprising the first isolates with Enterobacterales growth, was determined as n = 2889.

### Microbiological Methods

Isolate identification and antimicrobial susceptibility testing (AST) were primarily performed using the VITEK 2 automated system (bioMérieux, Marcy-l’Étoile, France). When required for identification, manual biochemical tests (IMViC: indole, methyl red, Voges–Proskauer, citrate) were applied; for AST verification or when deemed necessary, the disk diffusion method (Oxoid, Thermo Fisher Scientific, Basingstoke, UK) was used. Colistin susceptibility was tested only in carbapenem-resistant isolates (n = 40) using a broth microdilution-based Sensititre panel (Thermo Fisher Scientific, Cleveland, OH, USA). All results were interpreted according to the European Committee on Antimicrobial Susceptibility Testing (EUCAST) breakpoints in effect at the time of testing.

### Antimicrobial Categories

The following agents were analyzed under the indicated classes: penicillins (ampicillin); β-lactam/β-lactamase inhibitor combinations (amoxicillin-clavulanate, ampicillin-sulbactam, piperacillin-tazobactam); second-generation cephalosporins (cefuroxime, cefoxitin); third-generation cephalosporins (cefotaxime, ceftriaxone, cefixime, ceftazidime); fourth-generation cephalosporins (cefepime); monobactam (aztreonam); carbapenems (ertapenem, imipenem, meropenem); fluoroquinolones (ciprofloxacin, levofloxacin, moxifloxacin); aminoglycosides (amikacin, gentamicin); trimethoprim-sulfamethoxazole (TMP-SMX); nitrofurantoin; fosfomycin; glycylcycline (tigecycline); and amphenicol (chloramphenicol). For single-agent classes, class-level resistance equaled that agent’s resistance (e.g., monobactam = aztreonam; nitrofurantoin, fosfomycin, tigecycline, and chloramphenicol were each reported as single agents). All tested agents and per-agent denominators are provided in Supplementary Table 1. Because the number of colistin tests was low, colistin was not included in the heatmap analyses.

### Resistance Phenotypes and Operational Definitions

The analysis included *Escherichia coli*, *Klebsiella* spp., and *Proteus* spp. Intrinsic/inducible AmpC β-lactamase producers (*Enterobacter cloacae* complex, *Klebsiella aerogenes*) and the Others group (mostly *Citrobacter* spp., *Serratia* spp., *Morganella morganii*) were excluded from phenotype trend models. For susceptibility interpretation, intermediate (I) and resistant (R) results were combined as non-susceptible (NS). Multidrug-resistant (MDR), XDR, and PDR were defined according to the international consensus proposed by Magiorakos and colleagues.^8^ In this framework, MDR was defined as NS in at least 3 different antimicrobial classes; XDR as NS in all but at most 2 classes, provided that the number of classes tested was ≥6; and PDR as NS to all classes (and to all agents tested within those classes), provided that the number of classes tested was ≥8. Phenotypic confirmation for ESBL was not available; therefore, a study-specific classification was used: concurrent NS to at least one third-generation cephalosporin and to aztreonam was considered “ESBL-suspect.”^9^ Species-specific expected intrinsic resistances were interpreted based on the EUCAST Expected Resistant Phenotypes document;^10^ accordingly, ampicillin for *Klebsiella* spp., and nitrofurantoin and polymyxins for Proteeae were excluded from relevant summaries.

### Statistical Analysis

Analyses were conducted using IBM SPSS Statistics v25.0. Categorical variables were summarized as counts and percentages; group differences were assessed using the chi-square test or Fisher’s exact test when any expected cell count was <5. Continuous variables are reported as median (interquartile range, IQR). Temporal trends for 2020-2024 were evaluated using the Cochran–Armitage trend test and weighted logistic regression. Effect sizes were reported as odds ratio per year (OR/year) with 95% confidence intervals (CI). The Benjamini–Hochberg procedure was applied for multiple testing. Statistical significance was set at *P* < .05 (or q < .05). Because the data for 2025 were partial, trend analyses were restricted to 2020-2024.

### Ethics Approval and Informed Consent

This study was approved by the Clinical Research Ethics Committee of Training and Research Hospital (Approval No. 12-14, September 10, 2025). As this study was retrospective and used anonymized laboratory data, the requirement for informed consent was waived by the same ethics committee.

## Results

### Sample and Species Distribution

Between January 2020 and August 2025, *Enterobacterales* growth was detected in 2889 of 3359 positive urine cultures from pediatric patients (86.0%). Females constituted 72.3% of patients (n = 2088), males 27.7% (n = 801), and the median age was 2.0 years (IQR 0.3-6.0). The most frequently isolated species was *E. coli*, accounting for 69.4% of *Enterobacterales* isolates (n = 2,005), followed by *Klebsiella* spp. (20.6%, n = 595), *Proteus* spp. (5.6%, n = 163), and *Enterobacter* spp. (2.1%, n = 62). Less frequently, *Citrobacter* spp. (1.0%, n = 28), *Morganella morganii* (0.5%, n = 15), *Serratia* spp. (0.6%, n = 18), *Raoultella ornithinolytica* (n = 2), and *Salmonella* spp. (n = 1) were recovered.

### Clinical Units and Age Groups

Most specimens originated from General Pediatrics (88.0%, n = 2543), followed by Pediatric Nephrology (7.9%, n = 227), Neonatology (2.1%, n = 60), Pediatric Surgery (0.8%, n = 23), and Pediatric Gastroenterology (0.6%, n = 18); other units comprised 0.6% (n = 18). Of all isolates, 99.3% (n = 2869) were obtained from outpatients. *Escherichia coli* predominated across all age strata. As shown in Table 1, *Klebsiella* spp. were relatively more frequent in the neonatal (0-28 days) and 29 days-23 months age groups, and *Proteus* spp. were relatively more frequent in the 2-5 year group. For *Enterobacter* spp., the difference was statistically significant, mainly due to the higher proportion observed in the neonatal group.

### Species-by-Agent Resistance

Species-by-agent resistance rates are presented in Table 2. In *E. coli*, resistance to ampicillin was high (62.4%), whereas resistance to urinary agents was low (nitrofurantoin 2.9%, fosfomycin 9.4%). Compared with *E. coli*, *Klebsiella* spp. and *Enterobacter* spp. exhibited higher resistance to third- and fourth-generation cephalosporins and to carbapenems. Overall carbapenem resistance remained low, and susceptibility to amikacin and to urinary agents was largely preserved. Species–agent intrinsic mismatches are indicated in the table footnotes.

### Class-Level Resistance Summary

**Figure 1.**
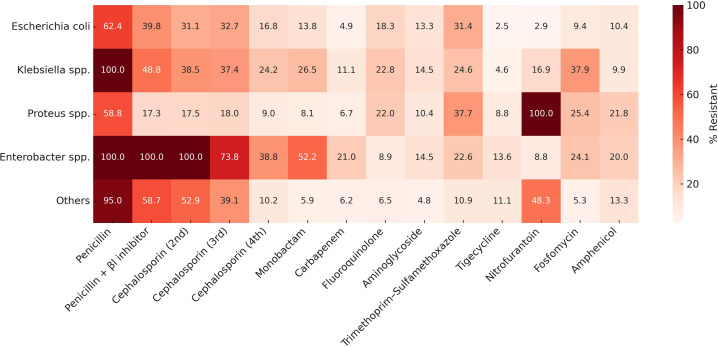
Class-level resistance heatmap for pediatric Enterobacterales urinary isolates. Horizontal axis, antibiotic classes; vertical axis, organisms. Color intensity reflects resistance percentage. Single-agent classes equal the corresponding agent. Intrinsic resistances are displayed as 100%.

Class-level resistance patterns are depicted as a heatmap in Figure 1. In *E. coli*, resistance was moderate to high for penicillins and third-generation cephalosporins, and low for carbapenems and urinary agents (nitrofurantoin, fosfomycin). In *Klebsiella* spp., resistance to third- and fourth-generation cephalosporins and to carbapenems was higher than in *E. coli*. In *Enterobacter* spp., resistance to third-generation cephalosporins and to the monobactam aztreonam was pronounced.

### Temporal Trends in Multidrug-Resistant, Extensively Drug-Resistant, Pandrug-Resistant, and Extended-Spectrum Beta-Lactamase-Suspect Phenotypes

**Figure 2.**
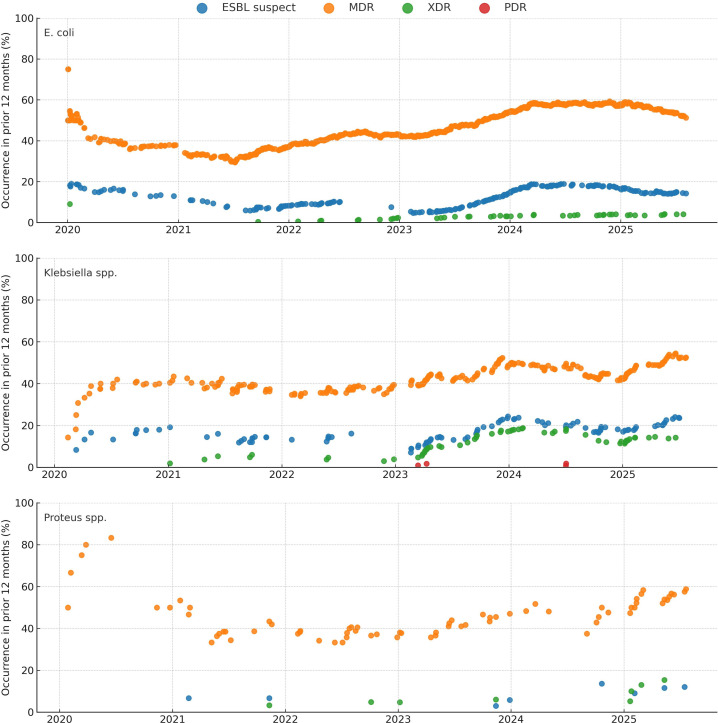
Temporal distribution of extended-spectrum beta-lactamase-suspect, multidrug-resistant (MDR), extensively drug-resistant (XDR), and pandrug-resistant among pediatric *Escherichia coli*, *Klebsiella spp.*, and *Proteus spp.* urinary isolates (2020-2025). Each point represents the proportion of the indicated phenotype among all isolates of the same species in the preceding 12 months. Colors denote phenotypes as shown in the legend (ESBL-suspect, blue; MDR, orange; XDR, green; PDR, red). Phenotypes were defined as specified in the Methods.

Temporal changes in phenotypes are shown in Figure 2. For *E. coli* (n = 2,005), MDR increased from 37.7% to 57.8%, ESBL-suspect from 12.0% to 17.5%, and XDR from 1.5% to 3.1% (Cochran–Armitage and weighted logistic regression; OR/year 1.29 [1.19-1.38], 1.22 [1.09-1.37], 1.57 [1.18-2.10], respectively; all q < .01). For *Klebsiella* spp., XDR increased (6.3% to 11.4%; OR/year 1.62 [1.23-2.13]; q = .002), and rare PDR increased (0.2% to 0.9%; OR/year 2.12 [1.21-3.73]; q = .018). No significant annual change was detected for MDR (43.9%) or ESBL-suspect (16.8%) in *Klebsiella* spp. (q > .05). For *Proteus* spp., no consistent annual trend was observed (all q > .05). Trend tests were conducted using 2020-2024 data.

### Class-Level Resistance Within Subgroups

**Figure 3.**
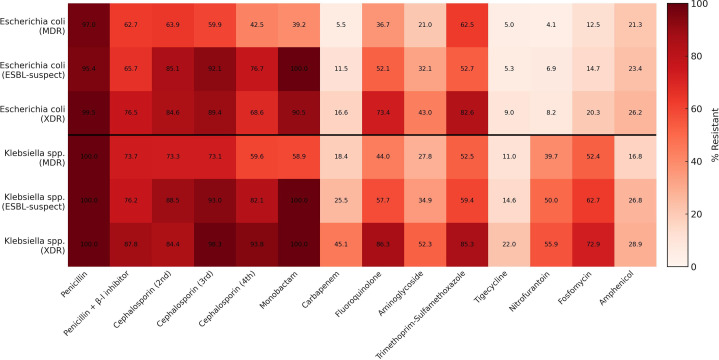
Heatmap of class-level resistance in ESBL-suspect, multidrug-resistant (MDR), and extensively drug-resistant subgroups of *Escherichia* *coli *and *Klebsiella *spp. Cells depict resistance percentages by antibiotic class; darker shading indicates higher resistance. Intrinsic resistances appear as 100%.

Figure 3 summarizes class-level resistance within ESBL-suspect, MDR, and XDR subgroups for *E. coli* (MDR n = 947, ESBL-suspect n = 240, XDR n = 47) and *Klebsiella* spp. (MDR n = 253, ESBL-suspect n = 97, XDR n = 56). In ESBL-suspect subgroups, resistance across β-lactams was high, accompanied by increased resistance to fluoroquinolones, aminoglycosides, and trimethoprim–sulfamethoxazole. In the *E. coli* MDR subgroup, susceptibility to carbapenems and urinary agents was largely preserved, whereas the XDR subgroup showed marked resistance to fluoroquinolones and aminoglycosides. In the *Klebsiella* spp. XDR subgroup, resistance to carbapenems and aminoglycosides was high.

## Discussion

This study evaluated species distribution, antibiotic susceptibility patterns at the class level, and temporal trends of MDR, XDR, and ESBL-suspect phenotypes in pediatric urinary *Enterobacterales* infections over a period exceeding 5 years, using a large cohort from a tertiary pediatric center in southern Türkiye. The primary aim was to translate local resistance surveillance into clinical decision-making and provide a foundation for updating empirical therapy protocols. The analysis demonstrated a consistent increase in MDR among *E. coli* and a significant rise in XDR in *Klebsiella* spp. These findings align with the global trend of increasing multidrug resistance in pediatric populations^2^^-^^4^ and suggest that local rates have reached or exceeded the upper range reported in national studies.^7^^,^^11^ The observed annual trends highlight the rapid dissemination of antimicrobial resistance and underscore the need for long-term surveillance in similar settings.^12^

Across age groups, *E. coli* remained the predominant species, although *Klebsiella* spp. was significantly more frequent in the neonatal (0-28 days) and 29 days-23 months age groups. In previous studies, this finding was commonly associated with neonatal intensive care colonization.^13^ However, since 99.3% of isolates in our study were community-derived and our center lacks a pediatric intensive care unit, this increase is unlikely to be hospital-acquired. Although causal inference was not possible due to the absence of individual-level data, early colonization, perinatal factors, and recent antibiotic exposure have been proposed as contributing factors.^14^^,^^15^ In the 2-5 year age group, a modest increase in *Proteus* spp. was noted, which may relate to male sex, lack of circumcision, and structural urinary anomalies that predispose to *Proteus* infections.^16^ Clinically, *Proteus* typically remains susceptible to most agents used empirically for *E. coli*, though its intrinsic resistance to nitrofurantoin and colistin should be considered.^17^ These findings suggest that age-specific pathogen distribution should inform empirical treatment strategies, with *Klebsiella* considered in infancy and *Proteus* in preschool-age infections.

Following the assessment of species distribution and age effects, the analysis focused on resistance phenotypes of the predominant species, *E. coli*. Among *E. coli* isolates, MDR was 47.2% (947/2005), ESBL-suspect 12% (240), and XDR 2.3% (47). Compared to pediatric reports showing broad ranges (MDR 32-88.2%, ESBL 24.6-64.3%, XDR 5-18.2%),^2^^-^^4^ our data indicate a relatively high MDR level but lower ESBL and XDR frequencies. In a multicenter Turkish pediatric study, the MDR rate in *E. coli* was 35.7%.^11^ Our higher MDR level likely reflects community-acquired resistance given the large cohort, extended study period, and predominantly outpatient origin of isolates. Differences in prescribing practices, age distribution, and healthcare access may also contribute.^6^^,^^18 ^

The trend analysis corroborates this high MDR tendency. Over 2020-2024, all resistance phenotypes increased significantly, with MDR showing an annual odds ratio (OR/year) of approximately 1.29. Under a log-linear model, this suggests MDR prevalence could double roughly every 3 years. Similarly, a Vietnamese study of pediatric *E. coli* isolates^19^ reported an increasing number of resistance genes between 2018 and 2020, including *aadA2* (aminoglycoside) and *blaNDM-5* (carbapenemase). Studies from Bangladesh^18^ and Peru^20^ also reported high MDR rates, indicating a global escalation of resistance among pediatric urinary isolates. However, long-term trend analyses in children remain scarce,^12^ making our findings a valuable contribution to this gap.

Within resistance phenotypes, the MDR subgroup exhibited high resistance to β-lactams, TMP-SMX, and fluoroquinolones, while carbapenem and amikacin susceptibility remained largely preserved. This pattern is consistent with the classical pediatric MDR *E. coli* profile.^7^ The preserved susceptibility to nitrofurantoin and fosfomycin supports their continued use as first-line options for lower urinary tract infections.^21^^,^^22^ In the XDR subgroup (n = 47), treatment options were markedly limited and must be guided by isolate-based testing.

When interpreting ESBL rates, it should be noted that the “ESBL-suspect” definition in our study was based on concurrent non-susceptibility to at least one third-generation cephalosporin and aztreonam, without phenotypic confirmation. This pragmatic approach has been suggested for large-scale resistance studies lacking confirmatory testing,^9^ though it may underestimate the true ESBL prevalence.

Taken together, the rising MDR burden and annual increase observed for *E. coli* highlight the need for institutional updates to empirical therapy algorithms and ongoing surveillance of resistance trends. Our findings support rational implementation of carbapenem-sparing strategies and emphasize the importance of preserving nitrofurantoin and fosfomycin efficacy through targeted stewardship.^16^^,^^23^^,^^24^

Following *E. coli*, *Klebsiella* spp. isolates demonstrated a particularly concerning resistance profile, especially in the XDR subgroup. The significant increase in XDR (6.3% to 11.4%; OR/year = 1.62) indicates a narrowing therapeutic window due to high resistance across multiple antibiotic classes. In pediatric *Klebsiella* isolates, XDR trends have been linked to widespread ESBL production and selective antibiotic pressure.^25^^,^^26^ The XDR subgroup exhibited high resistance to carbapenems and aminoglycosides, while relatively preserved susceptibility to agents with limited pediatric use (tigecycline, chloramphenicol) underscores the selective impact of antibiotic exposure. However, tigecycline achieves poor urinary concentrations, and chloramphenicol is limited by safety concerns.^27^^,^^28^ Pandrug-resistant (PDR) phenotype was detected only in 3 *Klebsiella* isolates and was absent in other species, suggesting sporadic occurrence. The escalation of carbapenem resistance from 18% in MDR to 45% in XDR highlights the progressive expansion of the resistance spectrum. Therefore, in high-risk cases, empiric therapy should be minimized in favor of early targeted treatment guided by PK/PD principles and rapid de-escalation once culture results are available.

*Proteus* spp. was identified as another clinically relevant species, with MDR 44.2%, ESBL-suspect 4.9%, and XDR 4.9%, without a significant temporal trend. Due to intrinsic resistance, nitrofurantoin and colistin are inappropriate for this species.^10^^,^^17^ High resistance to ampicillin and TMP-SMX has been attributed to plasmid-mediated β-lactamases and folate pathway resistance mechanisms.^29^ However, the lower frequency of ESBL and carbapenemase production compared to *E. coli* and *Klebsiella* spp. explains the relatively preserved susceptibility across several antibiotic classes.^30^ Clinically, in uncomplicated *Proteus* infections, ampicillin and TMP-SMX should be avoided, while second- or third-generation cephalosporins and amoxicillin–clavulanate may be appropriate empiric choices. In febrile or upper-tract infections, parenteral therapy with early de-escalation guided by culture results is recommended.^31^

Although *Enterobacter* spp. were infrequent, their resistance profile reflected expected intrinsic patterns. High resistance to ampicillin, amoxicillin–clavulanate, ampicillin–sulbactam, and cefoxitin indicates the presence of AmpC-mediated intrinsic and inducible β-lactamases.^23^ Higher resistance to third-generation cephalosporins and aztreonam, but lower resistance to cefepime, matches the class differentiation typical of AmpC producers.^23^ Elevated ertapenem resistance compared with imipenem and meropenem supports the differential carbapenem susceptibility described in the literature.^32^ These features emphasize the need for cautious interpretation in empirical selection. The variable but partially preserved susceptibility to urinary agents underscores the necessity of culture-guided therapy. In AmpC-producing species, third-generation cephalosporins are unreliable, and cefepime or appropriate carbapenems remain preferred options in severe cases.^23^^,^^32^

The “Others” group included *Citrobacter* spp., *Morganella morganii*, *Serratia* spp., *Raoultella ornithinolytica*, and a single *Salmonella* isolate. Due to small sample sizes and interspecies heterogeneity, trend analyses were not performed, and broad conclusions were avoided. Species-specific intrinsic phenotypes should guide interpretation; for example, *Proteeae* are intrinsically resistant to nitrofurantoin and polymyxins, while some *Serratia* and *Citrobacter* species are resistant to early-generation β-lactams.^10^^,17^ Clinical management should therefore be based on infection severity, with rapid adjustment once culture and susceptibility data are available. Local antibiograms, particularly for less common species, are critical for evidence-based decision-making.^31^

This study was not designed to investigate causal factors of antimicrobial resistance; however, several explanations proposed in the literature may help contextualize the observed resistance patterns. Increasing resistance to cephalosporins, penicillins, aminopenicillins, and other antibiotic classes has been primarily attributed to widespread and inappropriate antibiotic use, the expanding dissemination of resistance mechanisms such as ESBLs, repeated treatment courses, and the establishment of community reservoirs of resistant bacteria.^14^^,^^15^^,^^31^ These factors collectively emphasize the importance of incorporating local resistance patterns and antimicrobial stewardship principles into empirical treatment decisions. Despite this unfavorable resistance landscape, susceptibility to nitrofurantoin, a commonly used oral agent for uncomplicated urinary tract infections, and to amikacin, a frequently utilized parenteral option, remained largely preserved in our dataset. This finding is consistent with existing literature and supports the continued consideration of these agents as empirical treatment options when clinically appropriate.^7^^,^^21^

In summary, this 5-year pediatric cohort comprehensively characterized species distribution and resistance dynamics among urinary *Enterobacterales* isolates in children. The findings demonstrate an increasing MDR burden in *E. coli*, a significant rise in XDR among *Klebsiella* spp., and preserved susceptibility to carbapenems, amikacin, nitrofurantoin, and fosfomycin. These trends emphasize the need for age- and species-specific updates to empirical therapy protocols, periodic resistance monitoring, and careful continuation of carbapenem-sparing policies. This study provides one of the few long-term pediatric datasets documenting resistance trends, thereby contributing to national antimicrobial resistance surveillance efforts.

## Figures and Tables

**Table 1.** Distribution of Enterobacterales Species by Pediatric Age Groups

**Organism**	**0-28 days** **(n = 60)**	**29 days-23 months** **(n = 1.232)**	**2-5 years** **(n = 737)**	**6-11 years** **(n = 646)**	**12-18 years** **(n = 214)**	**Total** **(n = 2889)**	***P* value**
*Escherichia coli*	16 (26.7%)	725 (58.9%)	555 (75.3%)	549 (85.0%)	160 (74.8%)	2005	< .001
*Klebsiella* spp.	31 (51.7%)	381 (30.9%)	86 (11.7%)	58 (9.0%)	39 (18.2%)	595	< .001
*Proteus* spp.	1 (1.7%)	63 (5.1%)	73 (9.9%)	18 (2.8%)	8 (3.7%)	163	< .001
*Enterobacter* spp.	10 (16.7%)	31 (2.5%)	11 (1.5%)	9 (1.4%)	1 (0.5%)	62	< .001
Others	2 (3.3%)	32 (2.6%)	12 (1.6%)	12 (1.9%)	6 (2.8%)	64	.54

*P*-values from chi-square tests.

**Table 2.** Species-Specific Antibiotic Resistance Rates (%) Among Pediatric Enterobacterales Urinary Isolates

**Antibiotic**	**E. coli**	**Klebsiella spp.**	**Proteus spp.**	**Enterobacter spp.**	**Others**
Ampicillin	62.4	100^a^	58.8	100^a^	95
Amoxicillin–Clavulanate	36.5	44.4	15.8	100^a^	61
Ampicillin–Sulbactam	27.4	39.8	10.8	100^a^	40
Piperacillin–Tazobactam	15.7	31.8	0	44.4	6.2
Aztreonam	13.8	26.5	8.1	52.2	5.9
Ertapenem	4.1	10.7	4.4	19.7	6.6
Imipenem	1	5.3	2.9	2.8	0
Meropenem	0.9	6.6	1.9	4.8	3.1
Ciprofloxacin	15.3	19.9	18.5	7.1	6.6
Levofloxacin	10.3	12.6	14.6	10	7.4
Moxifloxacin	15	20.7	17.9	19	6.2
Amikacin	5.4	5.8	2.5	3.4	1.6
Gentamicin	10.8	13.9	9.4	14.5	4.9
Nitrofurantoin	2.9	18.3	100^a^	9.7	50
Fosfomycin	9.4	38.6	25.8	24.1	5.6
Chloramphenicol	10.4	9.9	21.8	20	13.3
Colistin^b^	4.2	0	100^a^	33.3	66.7
Cefoksitin	17	22.1	12.5	100^a^	26.7
Cefuroxime	31.1	38.5	17.5	90.9	52.9
Cefotaxime	7.6	10.6	0	55.6	20
Cefiksim	31.6	34.6	17.2	89.6	36.6
Ceftriakson	22.3	33.2	8.8	63.9	28.1
Ceftazidime	27.3	35.3	11.5	60.3	31.5
Cefepim	16.8	24.2	9	38.8	10.2
Tigesiklin	2.5	4.8	9.1	15.8	11.1
Trimethoprim-Sulfamethoxazole	31.4	24.6	37.7	22.6	10.9

^a^Intrinsic resistance (per EUCAST expected phenotypes); values excluded from class-level summaries. ^b^Colistin was assessed only in carbapenem-resistant isolates; values reflect the tested isolates.

## Data Availability

The data that support the findings of this study are available on request from the corresponding author.
